# Poor sleep quality and associated factors among healthcare professionals at the University of Gondar Comprehensive Specialized Hospital, Northwest Ethiopia

**DOI:** 10.3389/fpsyt.2024.1225518

**Published:** 2024-05-02

**Authors:** Winta Tesfaye, Ayechew Adera Getu, Baye Dagnew, Alemu Lemma, Yigizie Yeshaw

**Affiliations:** ^1^ Department of Human Physiology, School of Medicine, University of Gondar, Gondar, Ethiopia; ^2^ Department of Psychiatry, School of Medicine, College of Medicine and Health Science, University of Gondar, Gondar, Ethiopia; ^3^ Department of Epidemiology and Biostatistics, Institute of Public Health, University of Gondar, Gondar, Ethiopia

**Keywords:** poor sleep quality, Pittsburgh Sleep Quality Index, depression, health care professionals, Ethiopia

## Abstract

**Background:**

Poor sleep quality is linked to physiological dysfunction, which increases the risk of obesity, cardiovascular disease, cognitive impairment, and other medical conditions. Despite the known health risks of sleep disturbances, literature is still scant regarding sleep quality and its associated factors among healthcare professionals in Ethiopia. Therefore, this study aimed to determine the prevalence of poor sleep quality and its associated factors among healthcare professionals at the University of Gondar Comprehensive Specialized Hospital.

**Methods:**

An institution-based cross-sectional study was conducted at the University of Gondar Comprehensive Specialized Hospital, Northwest Ethiopia. A total of 418 healthcare professionals participated in the study. The study participants were chosen using the stratified random sampling method. Data were collected using a structured, self-administered questionnaire. The Pittsburgh Sleep Quality Index (PSQI) was used to assess the sleep quality of participants. Bi-variable and multivariable logistic regression models were used. p ≤ 0.05 was used to declare statistically significant variables.

**Results:**

The mean age of the study participants was 30.7 years (SD ± 6.3). The overall prevalence of poor sleep quality was 58.9% [95% CI (54.2, 63.6%)]. Being female [adjusted odds ratio (AOR) = 1.9, 95% CI (1.2, 2.9)], being a shift worker [AOR = 5.7, 95% CI (2.3, 14.3), not performing regular exercise [AOR = 2.08 (1.2–3.6)], being a khat chewer [AOR = 3.1, 95% CI (1.2, 7.6)], and having depressive symptoms [AOR = 2.6, 95% CI (1.3, 6.8)] were significantly associated with higher odds of having poor sleep quality.

**Conclusion:**

The prevalence of poor sleep quality among healthcare professionals was found to be high. As a result, we recommend that healthcare providers at the University of Gondar Comprehensive Specialized Hospital focus on early regular screening for sleep disturbances and pay special attention to shift work schedules and behaviors such as khat chewing, exercise, and depressive symptoms.

## Introduction

Sleep quality is described as the feeling of a sleep experience, incorporating components of sleep initiation, maintenance, quantity, and enjoyment upon awakening (1). According to the American Academy of Sleep Medicine, an adult should sleep approximately seven or more hours per day (2). Poor sleep quality is closely linked to an increased susceptibility to a broad range of disorders, ranging from poor vigilance and memory to reduced mental and physical reaction times, reduced motivation, depression, insomnia, metabolic abnormalities, obesity, immune impairment, and even greater risk of cancer and cardiovascular disorders (3, 4). It is also linked to lower productivity and an increased chance of workplace injury (5, 6).

Worldwide, the prevalence of sleep problems ranges from 1.6% to 56.0% (7–9). Sleep problems are also common among healthcare professionals and significantly affect their quality of life, productivity, and ability to do their work (10). The prevalence of poor sleep quality among health professionals was 56.3% in Turkey (11), 73.8% in Vietnam (12), 86.8% in Malaysia (13), 42.3% in Saudi Arabia (14), 85.9% in Riyadh (10), and 54.2% in Nigeria (15). It is also estimated to be 53% in our country, Ethiopia (16). As a result, disturbed sleep or interrupted circadian rhythms may also initiate pathological condition in the human body (17). Additionally, factors including sex (18, 19), age (18, 20, 21), shift work (15, 22–24), coffee consumption (13), khat chewing (25, 26), alcohol consumption (20), depression (27, 28), not engaging in regular exercise (29–31), self-rated health (32), and extra use of smartphones are also among the most common risk factors of disturbed sleep–wake cycle (17).

Poor sleep quality can cause medical problems like fatigue and exhaustion, as well as psychological ones like increased irritation and loss of attention. These difficulties can impede communication and coordination among healthcare workers (29, 33, 34). Consequently, it is critical to investigate sleep quality and associated factors among healthcare workers (HCWs) in diverse settings in order to reduce the impact of sleep disorders and medical errors. Additionally, Ethiopian healthcare policy does not emphasize healthcare practitioners’ sleep quality (35). Despite the well-known link between sleep disturbances and a variety of severe health problems, research on sleep quality in Ethiopian healthcare professionals is limited (16, 36).

As a result, the current study is meant to fill this gap. Therefore, this study aimed to assess the prevalence of poor sleep quality and associated factors among healthcare professionals at the University of Gondar Comprehensive Specialized Hospital, Northwest Ethiopia.

## Materials and methods

### Study setting

The study was conducted at the University of Gondar Comprehensive Specialized Hospital Northwest Ethiopia from February 1 to March 30, 2020. The University of Gondar Comprehensive Specialized Hospital is one of the biggest teaching hospitals in the Amhara region, serving over five million people requiring tertiary care. It is situated 728 km north of Ethiopia’s capital city, Addis Ababa. The hospital provides both comprehensive and referral-level treatment. There are 938 full-time registered healthcare workers. This research’s study population included all healthcare professionals working in the hospital.

### Study design and population

An institution-based cross-sectional study was carried out among all healthcare workers who were permanently employed at the University of Gondar Comprehensive Specialized Hospital and were available during data collection. During the data collection time, healthcare professionals who were unable to communicate due to a serious illness were excluded.

### Sample size determination and sampling procedure

The sample size for this study was determined using a single population proportion formula by assuming the prevalence of poor sleep quality among healthcare professionals (p = 50%), 95% confidence interval, and 5% margin of error. After adding a non-response rate of 10%, the final sample size became 423. There were a total of 876 healthcare professionals. Therefore, a stratified random sampling technique was applied based on their profession. First, a proportional allocation was used after identifying the number of healthcare workers in each profession. The study subjects were then chosen to form a total of 423 healthcare professionals using a computer-generated simple random sampling method from those strata.

### Data collection tools and technique

Data were gathered from February 1 to March 30, 2020, using a self-administered questionnaire that included socio-demographic variables, work-related factors, substance use, depression (as assessed by the Beck’s Depression Inventory), and the Pittsburgh Sleep Quality Index (PSQI). The PSQI is a validated tool used to assess adult sleep quality and patterns (37). Subjective sleep quality, sleep latency, sleep duration, habitual sleep efficiency, sleep disturbances, medication use, and daily dysfunction are the seven groups of the instrument. The seven domains are then added to create a total score, with a score greater than 5 indicating “poor” sleep quality and a score of less than or equal to 5 indicating “good” sleep quality (38).

### Study variables

Poor sleep quality was the dependent variable in this research. Socio-demographic variables (age, gender, marital status, having children, and profession), substance use variables (alcohol consumption, cigarette smoking, drinking coffee, and khat chewing), work-related variables (working shift, working hour, and work experience), and health status variables (depression, history of chronic illness, exercise, and self-rated health) are independent variables. To assess depression, we used the Beck Depression Inventory (BDI), which is a 21-item self-report rating assessment that assesses depression-related attitudes and symptoms (39). The Beck Depression Inventory, second edition (BDI-II) is one of the most widely used tools in research and application to assess the existence and degree of depression in the previous 2 weeks (40). The BDI-II assesses 21 symptoms and attitudes, which include Mood, Pessimism, Sense of failure, Lack of satisfaction, Guilt feelings, Sense of punishment, Self-dislike, Self-accusation, Suicidal wishes, Crying, Irritability, Social withdrawal, Indecisiveness, Distortion of body image, Work inhibition, Sleep disturbance, Fatigability, Loss of appetite, Weight loss, Somatic preoccupation, and Loss of libido (41, 42). Participants with BDI-II scores ranging from 0 to 13 were deemed normal. Participants with BDI-II scores of 14–19, 20–28, or 29–63 were classified as having mild, moderate, or severe depression, respectively (39). In terms of substance use, participants were considered current substance users if they had used cigarettes, khat, alcohol, or coffee at least once in the month preceding the survey (43–45). Khat (*Catha edulis*) is an herbal trade-related cultivated plant growing in most parts of the world, particularly in Eastern Africa and Arab countries (46, 47). It contains psychoactive substances cathinone and cathine, which can cross the blood–brain barrier to enter and stimulate the brain (47–49). A semi-structured question “Did you have a previous history of chronic illness?” was used to evaluate the history of chronic illness. A “yes” answer was considered as having a history of chronic illness. The questionnaire assesses shift work by asking if they have day and night shift work; if they answer positively with “yes”, they were classified as having shift work. Shift work entails switching between day and night shifts. A healthcare shift worker is a healthcare professional who often works in a healthcare facility switching between day and night (50). Regular exercise is described as a subset of physical activity that is planned, structured, and repetitive, with the ultimate or intermediate goal of improving or maintaining physical health (51). According to WHO guidelines, 150–300 minutes of moderate-intensity physical exercise or 75–150 minutes of vigorous-intensity physical activity is recommended (52).

### Statistical analysis

After checking for the completeness and consistency of the collected data, the data were entered into Epi-data version 3.02 and exported to SPSS version 25 for analysis. To express descriptive results, frequency with percent and mean with standard deviation were computed. The study fulfilled all chi-square assumptions, which assume that both factors are categorical, all observations are independent, and cells in the contingency table are mutually exclusive; there should be no zero cell values, and the expected value of cells should be 5 or higher in at least 80% of cells. A binary logistic regression was performed to determine the crude association between each independent variable and poor sleep quality. Variables in the bi-variable analysis with a p-value <0.25 were candidates for multivariable binary logistic regression analysis. The crude odds ratio (COR) and adjusted odds ratio (AOR) with 95% CI were calculated. For the multivariable analysis, variables with a p-value <0.05 were considered statistically significant.

### Data quality management

To ensure the data quality, 1-day training was given for four BSc nursing data collectors. The questionnaire was also pretested, and regular supervision of the data collection process was also made to increase the completeness, accuracy, and consistency of the data. Incomplete questionnaires were discarded from the analysis. Cronbach’s alpha was also calculated to test the reliability of the PSQI tool, and it was 0.72.

### Ethical consideration

Ethical clearance was obtained from the Ethical Review Board of the University of Gondar with ethical clearance number 1840/2012. To ensure the confidentiality of respondents, informed written consent was obtained from each study participant, and their names were not written on the questionnaire, which reduced the authors’ access to information that could identify individual participants during or after data collection.

## Results

### Socio-demographic characteristics of healthcare professionals

This survey had 418 participants. Initially, the plan was to collect data from 423 professionals, but due to incomplete and non-returned questionnaires, the response rate became 98.8%. Two hundred thirteen (51%) were female. The subjects’ mean age was 30.7 years (SD 6.3). One-third (33.3%) of the individuals were married. More than half of the participants in the research were nurses, 226 (54.1%), with midwives accounting for the remaining 60 (14.4%) (**Table 1**).

**Table 1:** Socio-demographic characteristics of healthcare professionals working at the University of Gondar Comprehensive Specialized Hospital, Northwest Ethiopia, 2020 (n = 418).

Variables	Category	Frequency	Percentage
Age (in years)	20–30 31–40 41–50 >50	265 119 26 8	63.4% 28.5% 6.2% 1.9%
Sex	Male Female	205 213	49.1% 50.9%
Marital status	Single Married Divorced	261 139 18	62.4% 33.3% 4.3%
Number of children	0 1–2 3–4 >4	277 101 36 4	66.3% 24.1% 8.5% 1.1%
Working profession	Medical doctor Pharmacist Nurse Laboratory technologist Midwife Others*	27 35 226 46 64 20	6.5% 8.4% 54.0% 11.0% 15.3% 4.8%

*Anesthesiologist, psychiatrist, public health practitioner, and physiotherapist.

### Work- and health-related characteristics of the respondents

Three hundred eighty-five (92.1%) of the participants were on duty. Four hundred sixty (97.8%) of them worked fewer than 56 hours per week. In terms of substance use, 45 (10.8%), 260 (62.2%), and 35 (8.4%) of the subjects were current khat chewers, alcoholic drinkers, and cigarette smokers, respectively. Thirty-five (8.4%) of those who participated had a history of chronic disease. The majority of subjects (82.3%) reported moderate stress (**Table 2**).

**Table 2:** Work- and health-related characteristics of healthcare professionals working at the University of Gondar Comprehensive Specialized Hospital, Northwest Ethiopia, 2020 (n = 418).

Variables	Categories	Frequency	Percentage
Working in shift	Yes No	385 33	92.1% 7.9%
Work experience (years)	1–9 10–19 20–29 ≥30	310 81 22 5	74.2% 19.4% 5.3% 1.2%
Working hours per week	≤56 >56	409 9	97.8% 2.2%
Current khat chewing	Yes No	45 375	10.8% 89.2%
Current alcohol drinking	Yes No	260 158	62.2% 37.8%
Current cigarette smoking	No Yes	383 35	91.6% 8.4%
Drinking coffee	Yes No	306 112	73.2% 26.8%
History of chronic illness	Yes No	35 383	8.4% 91.6%
Regular exercise	Yes No	76 342	18.2% 81.8%
Self-rated health	Excellent Very good Good Fair	119 152 120 27	28.5% 36.4% 28.7% 6.5%
Depression	Normal Mild Moderate and above	323 47 27 21	77.3% 11.2% 6.5% 5%

### Prevalence of poor sleep quality among healthcare professionals

The overall prevalence of poor sleep quality among healthcare professionals in this study was 58.9% [95% CI (54.2, 63.6%)]. Participants’ average night sleep duration and sleep latency were 6.5 hours (SD 1.27) and 18.6 minutes (SD 16.6), respectively. Three hundred five (73%) of the participants had less than 7 hours of sleep per day. Thirty-three (7.9%) and 73 (17.5%) had low habitual sleep efficiency (<65%) and used sleep medication in the past month, respectively (**Table 3**).

**Table 3:** Sleep quality and its component scores among healthcare professionals working at the University of Gondar Comprehensive Specialized Hospital, Northwest Ethiopia, 2020 (n = 418).

Variables	Categories	Frequency	Percentage
Subjective sleep quality (score)	Very good (0) Fairly good (1) Fairly bad (2) Very bad (3)	158 129 61 70	37.8% 30.9% 14.6% 16.7%
Sleep latency (score)	Never (0) <1 times per week (1) 1–2 times a week (2) ≥3 times a week (3)	95 234 74 15	22.7% 56% 17.7% 3.6%
Sleep duration	>7 hours 6–7 hours 5–6 hours <5 hours	113 206 71 28	27% 49.3% 17% 6.7%
Sleep efficacy	>85% 75%–84% 65%–74% <65%	209 127 49 33	50% 30.4% 11.7% 7.9%
Sleep disturbance (score)	Never (0) <1 times a week (1) 1–2 times a week (2) ≥3 times a week (3)	6 290 118 4	1.4% 69.4% 28.2% 1%
Use of sleep medication (score)	Never (0) <1 times a week (1) 1–2 times a week (2) ≥3 times a week (3)	345 48 10 15	82.5% 11.5% 2.4% 3.6%
Daytime dysfunction (score)	Never (0) <1 times a week (1) 1–2 times a week (2) ≥3 times a week (3	121 185 95 17	28.9% 44.3% 22.7% 4.1%

### Associated factors of poor sleep quality

In the logistic regression analysis, variables with p-value <0.25 were included in the multivariable logistic regression model. Accordingly, sex, shift work, current khat chewing, depression, and not performing exercise were significantly associated with poor sleep quality (p < 0.05).

Female participants were two times [AOR = 1.9, 95% CI (1.2, 2.9)] more likely to experience poor sleep quality compared to male participants. Participants who worked in shifts were six times [AOR = 5.7, 95% CI (2.3, 14.3)] more likely to have poor sleep quality than their counterparts. Participants who did not have regular exercise were two times more likely to have poor sleep quality than those who had regular exercise. Current khat chewers were three times [AOR = 3.1, 95% CI (1.2, 7.6)] more likely to experience poor sleep quality than those who did not chew khat. Participants with moderately severe depression were three times more likely to have poor sleep quality than those without depression [AOR = 2.6, 95% CI (1.3, 6.8)] (**Table 4**).

**Table 4:** Factors associated with poor sleep quality among healthcare professionals working at the University of Gondar Comprehensive Specialized Hospital, Northwest Ethiopia, 2020 (n = 418).

Variables	Categories	Poor sleep quality		OR (95% CI)	
		Yes	No	COR	AOR
Sex	Male Female	102 (24.4%) 144 (39.4%)	103 (24.6%) 69 (16.5%)	1 2.1 (1.4–3.1)	1 1.9 (1.2–2.9)*
Shift working	Yes No	239 (57.2%) 7 (1.7%)	146 (34.9%) 26 (6.2%)	6.1 (2.5–14.3) 1	5.7 (2.3–14.3)* 1
Current khat chewing	Yes No	38 (9.1%) 208 (49.8%)	7 (1.7%) 165 (39.5%)	4.3 (1.8–9.8) 1	3.1 (1.2–7.6)* 1
Drinking coffee	Yes No	191 (45.7%) 55 (13.2%)	115 (27.5%) 57 (13.6%)	1.7 (1.1–2.6) 1	1.2 (0.7–2.04) 1
Self-rated health	Excellent Very good Good Fair	60 (14.4%) 81 (19.4%) 80 (19.1%) 25 (6%)	59 (14.1%) 71 (17%) 40 (9.6%) 2 (0.5%)	1 1.1 (0.6–1.8) 1.9 (1.1–3.3) 12.2 (2.7–59.2)	1 0.9 (0.5–1.7) 1.3 (0.7–2.4) 1.2 (0.4–3.1)
Regular exercise	Yes No	29 (6.9%) 217 (51.9%)	47 (11.2%) 125 (29.9%)	1 2.8 (1.6–4.6)	1 2.08 (1.2–3.6)*
Depression	Normal Mild Moderate to severe	175 (41.9%) 35 (8.4%) 36 (8.6%)	148 (35.4%) 12 (2.9%) 12 (2.9%)	1 2.4 (1.23–4.92) 2.1 (0.9–4.6)	1 2.1 (0.9–4.5) 2.6 (1.3–6.8)*

COR, crude odds ratio; AOR, adjusted odds ratio.

* p ≤0.05.

## Discussion

The current study aimed to determine the prevalence and risk factors for poor sleep quality among healthcare professionals at the University of Gondar Comprehensive Specialized Hospital. In this study, the prevalence of poor sleep quality was 58.9% [95% CI (54.2, 63.6%)], indicating that a significant number of participants are affected by the issue. This finding is in line with the results of studies conducted in Turkey (55.3%) (11), Malaysia (57.8%) (13), and Nigeria (54.2%) (15). Compared to previous studies in France (64.8%) (53), Mexico (56.7%) (28), Colombia (74.9%) (54), Saudi Arabia (73.4%) (55), Riyadh (85.9%) (10), China (75%) (56), Malaysia (86.8%) (13), and Ethiopia (70.6%) (57), this research discovered a lower prevalence of poor sleep quality among healthcare workers. Differences in study population could account for the disparity in prevalence of poor sleep quality among studies as a potential cause of this variation. Unlike the current study, which included participants from all healthcare professions, the previous studies only included nurses; this was the case in earlier Ethiopian and Chinese studies, as well as physicians in the Malaysian and Riyadh studies. Our study’s results outperform those of Nepal (48.03%) (58), Saudi Arabia (42.3%) (14), and Ethiopia (25.6%) (59). This disparity could be due to differences in the instrument used or differences in sample size. The previous research in Ethiopia, for example, used the shift work sleep disorder questionnaire to assess sleep quality, whereas the PSQI was used in this study.

The current research found sex, shift work, current khat chewing, depression, and a lack of regular exercise as determinants of poor sleep quality. Female gender was associated with an increased odds of poor sleep quality. A similar finding was reported from a study conducted in Austria (60), Pakistan (19), Spain (18), Saudi Arabia (61), and Ethiopia (62). This could be due to increased household and family responsibilities in women, which is typically associated with women working for extended periods of time at night, which could affect their sleep quality. Furthermore, in terms of the other differences in circadian timing between the sexes, it has been hypothesized that somewhat shorter circadian periods in women may cause them to be more out of circadian alignment, resulting in increased sleeplessness (63). This study also revealed that people who work in shifts have increased odds of poor sleep quality than non-shift workers. Consistent with the current study, studies in China (23), Spain (22), and Ethiopia (57) stated that people working in shifts had an increased risk of poor sleep quality than those working without shifts. A plausible explanation for this could be that working in a shift rotation could have an unpredictable working schedule that disrupts the circadian rhythm and restricts opportunities for sleep (15, 59).

As for the present study’s result, current khat chewers have demonstrated three times higher odds of poor sleep quality than non-khat chewers. This finding aligns with studies performed in Yemen and Ethiopia (25, 26, 62, 64). This could be due to the effect of khat. Because of its primary active ingredient (cathinone), khat (*C. edulis*) is initially a stimulant with effects similar to those of amphetamine (26, 65). However, this euphoric condition is typically followed by depression, irritability, anorexia, and sleeping difficulties. Cathinone’s effects are achieved by reduced dopamine uptake by nerve terminals, increased dopamine release, and inhibition of monoamine oxidase. As a result of the persistent stimulation of post-synaptic neurons following a high amount of dopamine in the synaptic cleft, all processes may result in poor sleep quality. After 8 hours of chewing, cathinone is scarcely detectable in the blood. The first-pass metabolism of cathinone in the liver results in the production of norepinephrine, which reduces sleep quality (20, 26, 66).

The present study found that participants who did not exercise regularly were more likely to have poor sleep quality, which is consistent with earlier research conducted in China and Brazil (29, 67, 68). This is because physical activity can produce positive changes in circadian rhythms and increase adenosine levels in the body, both of which help to regulate sleep (69). Furthermore, exercise promotes the production and release of melatonin, which frequently leads to improved sleep quality (70). This indicates that regular exercise, as recommended by WHO (71), is critical for reducing not only non-communicable diseases but also sleep problems, which is why the American Sleep Disorder Association recommends physical activity as a crucial non-medicinal intervention for sleep disorders (72).

The current research found a link between sleep quality and moderate-to-severe depressive symptoms. This result is congruent with research from Turkey, Saudi Arabia, China, and Ethiopia (27, 28, 73–75). This could be because people who suffer from depression have lower melatonin production and delays in the circadian rhythm of melatonin (76, 77). Another potential explanation is that in depressed people, nocturnal melatonin release is often reduced, which may be linked to sleep disturbances (78, 79).

### Limitations of the study

Our study is not free from limitations. Because the study is cross-sectional, we are unable to show a cause–effect relationship between independent variables and poor sleep quality. Additionally, we could not show the dose–response relationship between substance use and sleep quality. Another limitation of this research could be recall bias.

## Conclusion

The current study found that poor sleep quality was highly prevalent among healthcare providers at the University of Gondar Comprehensive Specialized Hospital and had significant associations with sex, shift work, khat chewing, exercise, and depressive symptoms. As a result, we recommend that healthcare providers at the University of Gondar’s Comprehensive and Specialized Hospital focus on early regular screening for sleep disturbances and pay special attention to shift work schedules and behaviors such as khat chewing, exercise, and depressive symptoms. Finally, we recommend other researchers perform additional investigations on factors like body mass index (BMI), sleep hygiene, and using the Internet, which have not been explored in the current research among healthcare professionals, because they have public health implications.

## Data availability statement

The raw data supporting the conclusions of this article will be made available by the authors, without undue reservation.

## Ethics statement

The studies involving humans were approved by the Ethical Review Board of University of Gondar. The studies were conducted in accordance with the local legislation and institutional requirements. The participants provided their written informed consent to participate in this study.

## Author contributions

WT conceived the study, coordinated the process of data collection, was involved in data cleaning and statistical analysis, and prepared the first draft of the manuscript. AA, BD, AL, YA, and YY supervised the data collection process and participated in statistical analysis and interpretation of the results, manuscript reviewing, and editing. All authors contributed to the article and approved the submitted version.
